# Conservative Management of a Delayed Neovesicocutaneous Fistula

**DOI:** 10.1155/2014/632917

**Published:** 2014-06-29

**Authors:** Koichi Kodama, Yasukazu Takase, Isamu Motoi

**Affiliations:** Department of Urology, Toyama City Hospital, 2-1 Imaizumi-hokubucho, Toyama, Toyama 939-8511, Japan

## Abstract

A neovesicocutaneous fistula is a rare complication after orthotopic bladder reconstruction, particularly in the late postoperative period. We report the case of a 59-year-old man who had undergone ileal neobladder construction 17 months previously. He presented with urinary retention concomitant with urinary tract infection due to a neovesicourethral anastomotic stricture. After a combination of transurethral catheter drainage and broad-spectrum antibiotic therapy for 3 weeks, the fistulous tract completely closed. Therefore, conservative treatment may be regarded as a valid option for a delayed neovesicocutaneous fistula.

## 1. Introduction

Although the use of detubularized ileum for total bladder reconstruction has gained wide acceptance, this easy-to-use substitute is associated with certain specific complications. A neovesicocutaneous fistula is a rare complication following an ileal neobladder construction. In most cases, it develops during the early postoperative period because of suture failure; in some cases, it may occur because of local tumor recurrence in the late postoperative period. In this report, we present a case of a delayed neovesicocutaneous fistula that was caused by overdistention and bacterial urinary tract infection, which was successfully managed with conservative treatment.

## 2. Case Presentation

A 59-year-old man presented with a 1-week history of abdominal distention, decreased urine output, and the body temperature of 39°C. He had undergone retroperitoneoscopic nephroureterectomy with open excision of the bladder cuff 22 months earlier for urothelial carcinoma (UC) of the right ureter. His kidney along with the ureter was removed using a median incision in the lower abdomen. Five months after his nephroureterectomy, radical cystoprostatectomy and orthotopic ileal neobladder construction were performed for UC of the bladder in 2011. According to the classification of TNM, his clinical stage was T2aN0M0. A reservoir was created with a detubularized ileal U-shaped neobladder using 45 cm of the terminal ileum as described by Koie et al. [1]. His postoperative course was uneventful. At 12 months after operation, he had no incontinence and enuresis, a bladder capacity of 400–500 mL, and a postvoid residual urine volume of <50 mL, without requiring clean intermittent catheterization.

**Figure 1:**
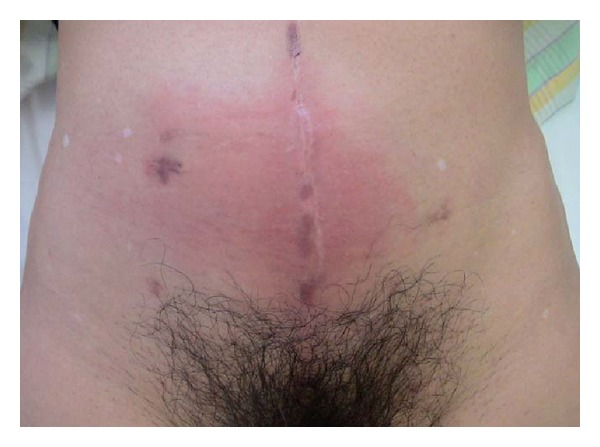
Images showing lower abdominal bulging with skin redness.

**Figure 2:**
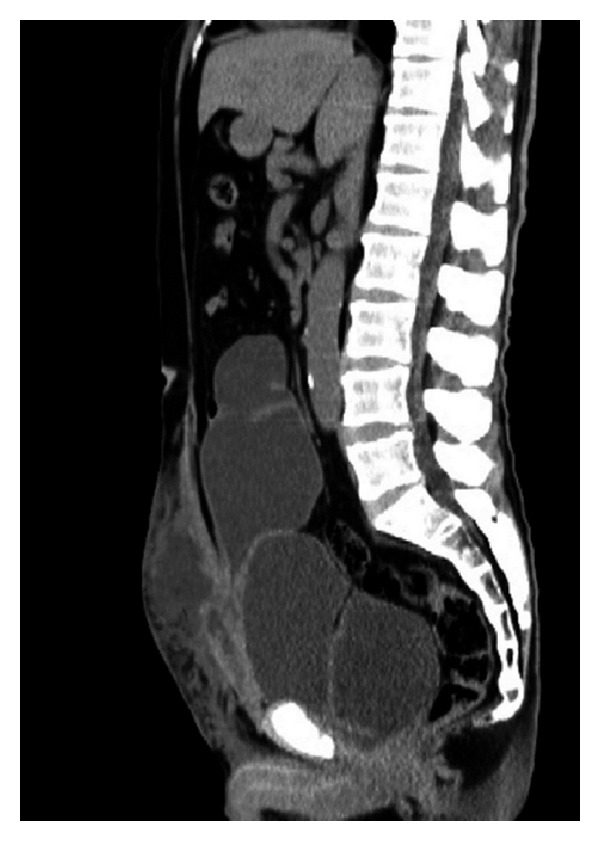
Sagittal unenhanced computed tomography images of the abdomen showing a distended neobladder and subcutaneous fluid accumulation in the lower abdomen. There is no evidence of ascites, intraperitoneal free gas, or incisional herniation.

A physical examination revealed lower abdominal bulging with skin redness (Figure 1). The laboratory findings were as follows: white blood cell count, 10,700/mm^3^; platelets, 200,000/mm^3^; serum creatinine, 2.15 mg/dL; and C-reactive protein, 30.74 mg/dL. Unenhanced computed tomography (CT) revealed a distended neobladder with left-sided hydroureteronephrosis and subcutaneous fluid accumulation in the lower abdomen (Figure 2). There was no evidence of ascites, intraperitoneal free gas, or incisional abdominal herniation. Flexible cystoscopy confirmed a neovesicourethral anastomotic stricture, which was treated by endoscopic urethrotomy. Subsequently, a wide-bore indwelling catheter was placed and 2,000 mL of turbid urine was collected.* Staphylococcus agalactiae* was isolated from the urine.

**Figure 3:**
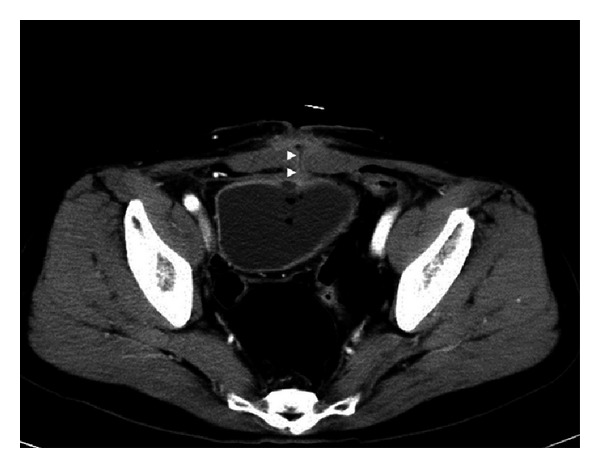
Axial contrast-enhanced computed tomography images of the abdomen showing a fistula (arrowheads) between the neobladder and skin, without apparent ischemia of the neobladder ileum.

**Figure 4:**
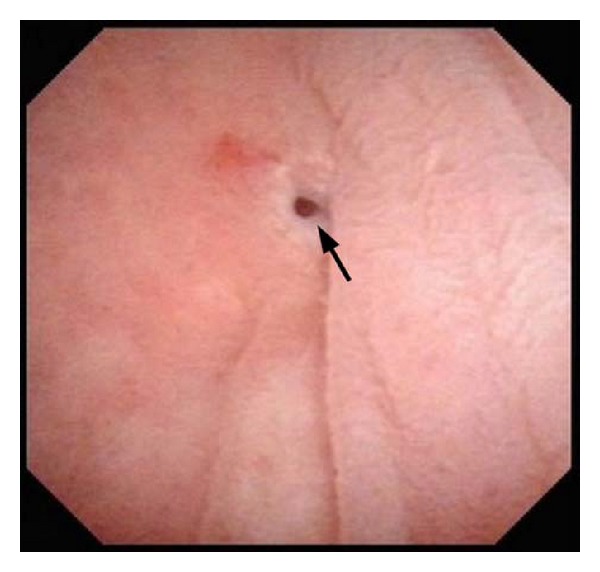
Flexible cystoscopy findings showing a pinhole (arrow) in the anterior wall of the ileal neobladder.

Seven days later, the renal function returned to normal, but a spontaneous rupture of the subcutaneous fluid into the median surgical scar occurred. The bladder was filled with indigo carmine-stained saline, which confirmed the formation of a neovesicocutaneous fistula. In addition, contrast-enhanced CT of the abdomen revealed that the fistula was between the neobladder and skin, without apparent ischemia of the neobladder ileum (Figure 3). Flexible cystoscopy revealed a pinhole in the anterior wall of the ileal neobladder (Figure 4). However, there was no evidence of local UC recurrence. Because he was clinically stable, a surgical exploration was deferred, and he underwent a conservative treatment with urethral catheter drainage and broad-spectrum antibiotics. Subsequently, the fistulous tract closed 3 weeks later.

After the urethral catheter was removed, he spontaneously voided every 4 h and had a postvoid residual urine volume of <50 mL, without requiring intermittent self-catheterization. The patient has been well without any recurrences of neovesicocutaneous fistula for 20 months after the fistula closure.

## 3. Discussion

Voiding dysfunction that results in urinary retention is a common complication of an orthotopic neobladder. In a large series study, 75 of the 655 male patients (11.5%) with orthotopic neobladders had at least one transient episode of urinary retention [2]. Urinary retention may be caused by infraneovesical obstruction or dysfunctional voiding. In another study of 923 patients with orthotopic neobladders, 3.1% developed infraneovesical obstruction; of these, 1.1% developed obstruction because of local tumor recurrences, 1.2% because of a neovesicourethral anastomotic stricture, and 0.9% because of a urethral stricture [3]. The risk factors for strictures at neovesicourethral anastomosis have not yet been determined. However, some of the risk factors for strictures at vesicourethral anastomosis in patients after radical prostatectomy may be relevant, including postoperative urine extravasation, asymptomatic bacteriuria, and extensive intraoperative blood loss.

Multiple adhesions can occur during neobladder healing in the decompressed state during the first 3 weeks after operation. These adhesions may create wall tension areas during filling, which eventually results in wall thinning and reduced elasticity. These can lead to a seromuscular tear of the bladder wall during overfilling. An orthotopic neobladder can open up to nearly all the surrounding pelvic structures, including the small bowel, colon, rectum, vagina, and iliac vessels. Therefore, the formation of fistulas in these structures has been reported, usually during the immediate postoperative period due to suture failure [4–6]. In contrast, the formation of a neovesicocutaneous fistula that occurs during the late postoperative period usually involves local tumor recurrence [3].

Damage to an orthotopic ileal neobladder plays an essential role in the formation of a delayed neovesicocutaneous fistula that is not caused by tumor recurrence. Mechanical damage may result from the rupture and perforation of an orthotopic ileal neobladder [7, 8]. Overdistention and bacterial urinary tract infection of the neobladder are usually associated with rupture and perforation, as observed in our case. Other factors associated with the weakening of the orthotopic bladder wall, including transmural infection and chronic ischemic changes in the bladder wall, are also thought to predispose patients to spontaneous perforation. Direct catheter trauma is believed to be an unlikely etiology because the rupture site is usually distant from the site of possible catheter injury.

The treatment algorithm for urinary fistulas of a neobladder initially involves adequate, uninterrupted urinary drainage and broad-spectrum antibiotics. Recently, cyanoacrylate glues were used to manage urinary fistulas, including neovesicocutaneous fistulas, with high success rates [9]. When these conservative maneuvers fail, fistulous tract occlusion can be performed because surgical repair may be challenging. Based on the excellent outcome of our present case, conservative management may be considered as a valid treatment option even for delayed neovesicocutaneous fistulas.

## 4. Conclusion

A neovesicocutaneous fistula is a rare complication that occurs after orthotopic bladder reconstruction, particularly during the late postoperative period. Adequate, uninterrupted urinary drainage and broad-spectrum antibiotic therapy may be a treatment option for this condition.
